# Supplementary material for: Synergistic targeting of cancer cells through simultaneous inhibition of key metabolic enzymes
Source: Cell Death Differ. 2025 Jun 23;32(12):2239–56. doi: 10.1038/s41418-025-01532-5 (PMC12669732; doi:10.1038/s41418-025-01532-5)
Supplement: Supplementary file 1 — Supplementary Data [file 41418_2025_1532_MOESM1_ESM.pdf]

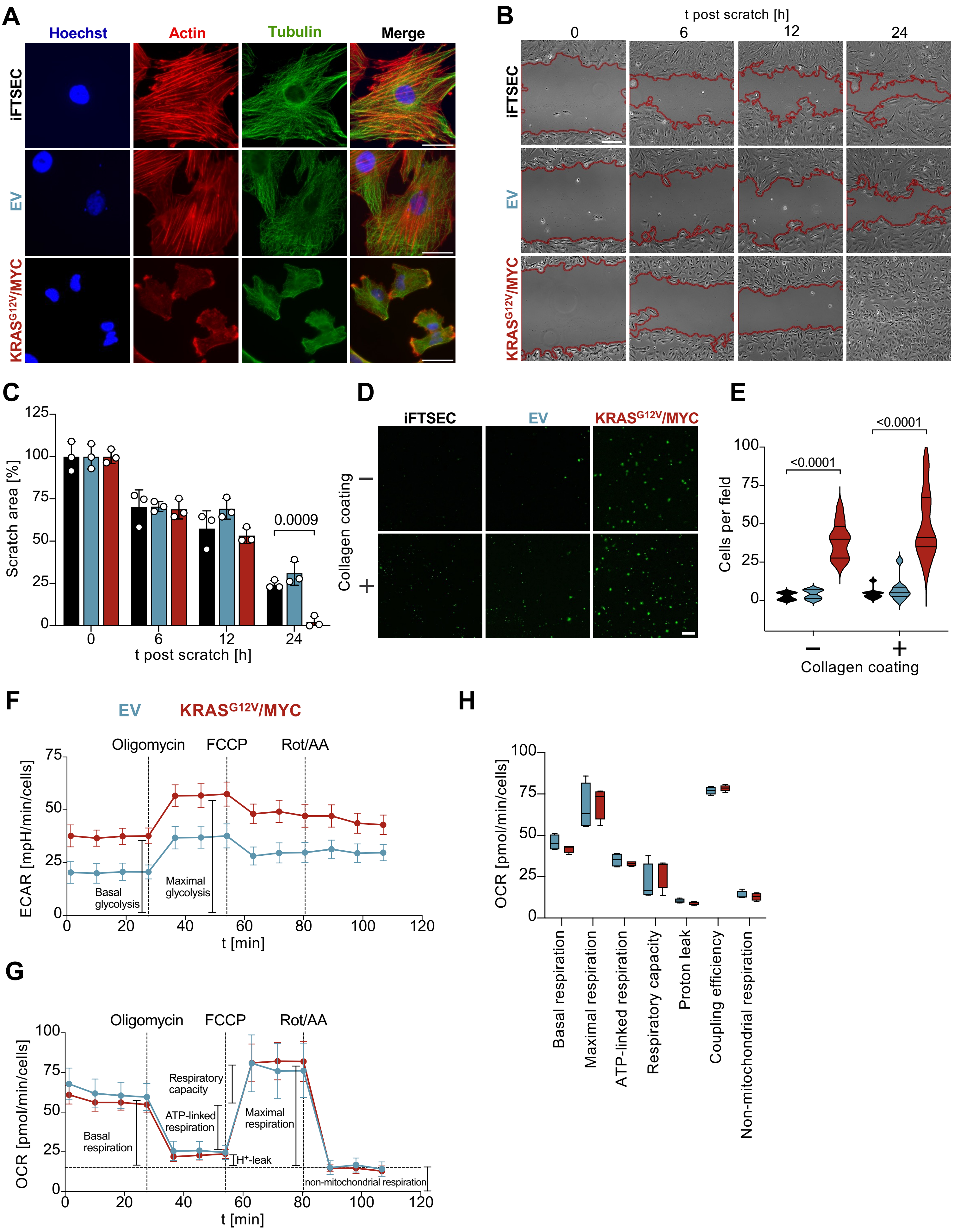

Supplementary Data Fig.1

Supplementary Data Fig. 1. Characterization of oncogenic transformation of KRAS^{G12V}/MYC cells.

(A) iFTSEC, EV and KRAS^{G12V}/MYC cells were stained for Actin and Tubulin by immunofluorescence microscopy. Representative examples are shown, the nuclear DNA was stained with Hoechst33342. Scale bar = 50 µm. **(B)** The indicated cells were grown to confluency and a scratch was made. Cells were further cultivated in medium containing only 1% (v/v) FCS to suppress proliferation. Closure of the scratch (indicated by red lines) was monitored over time. Scale bar = 200 µm. **(C)** Quantification of scratched areas. Shown are mean ± SD, *n* = 3, two-way ANOVA with Dunnett's multiple comparisons test, the control was set to 100% **(D)** Transwell assay without collagen (monitoring migration) and collagen coating (monitoring invasion). Scale bar = 200 µm. **(E)** Quantification of migrated or invaded cells per field. Shown are violin plots, *n* = 3, two-way ANOVA with Dunnett's multiple comparisons test. For the metabolic analysis displayed in the next subfigures, the indicated cell lines were analyzed by Seahorse metabolic flux analysis using the MitoStress test assay. At the indicated time points Oligomycin (2 µM), FCCP (2 µM) and Rotenone/Antimycin A (0.5 µM) were injected. **(F)** Analysis of glycolysis by measuring the extracellular acidification rate (ECAR). **(G)** Analysis of OXPHOS by measuring the oxygen consumption rate (OCR). **(H)** Quantification of different respiratory mechanisms. Shown are mean ± SD, *n* = 4, two-way ANOVA with Šídák's multiple comparisons test.

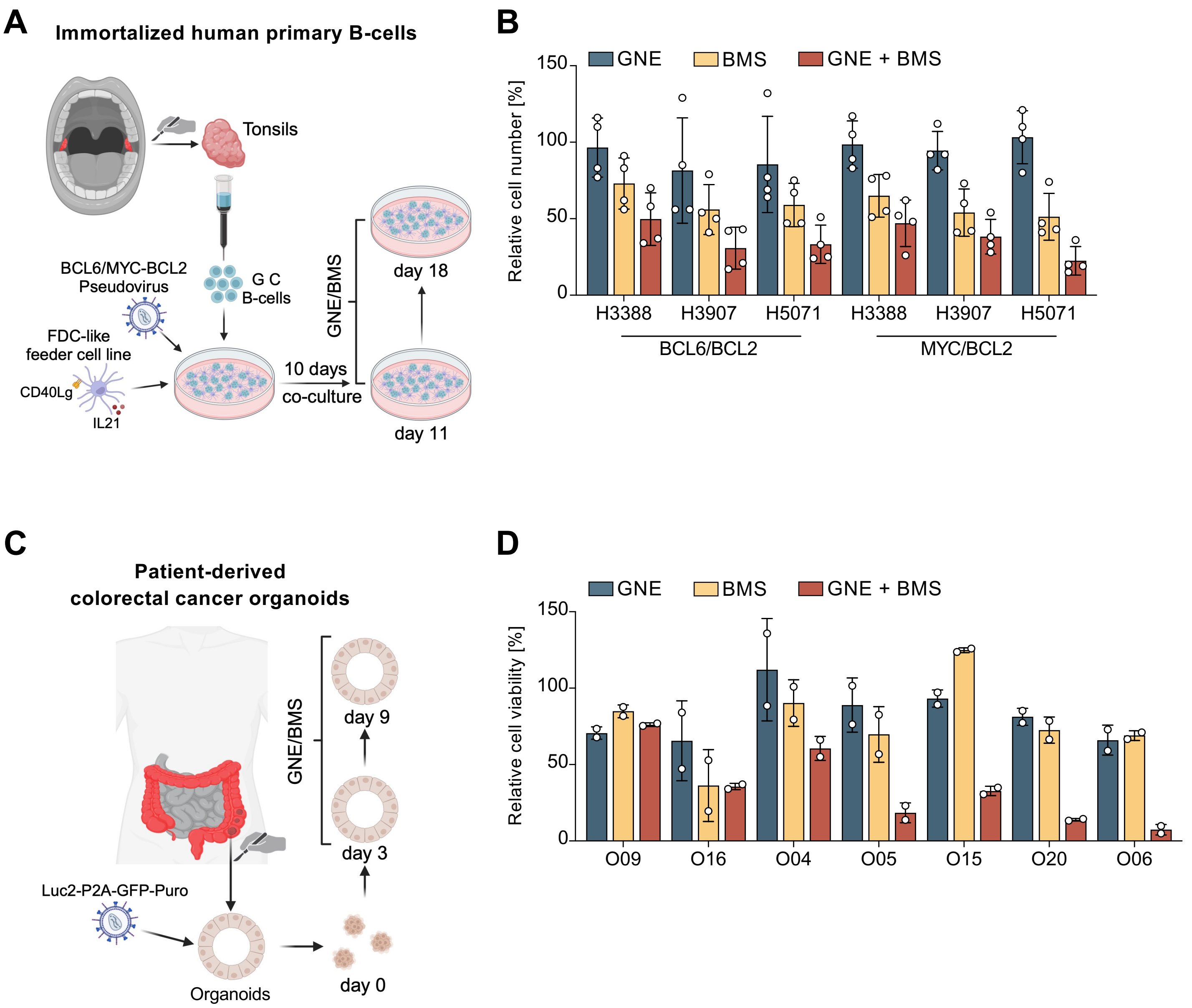

Supplementary Data Fig. 2. Impact of GNE/BMS treatment on human cell models.

(A) Primary human germinal center B cells were isolated from tonsils, immortalized by viral delivery of BCL2 with BCL6 or MYC and further grown on feeder cells, as schematically shown. Created in BioRender. Schmitz, L. (2025) https://BioRender.com/ikd4c2w. **(B)** Cells were sub-cultured for 10 days on irradiated FDC-like feeder cells prior treatment with sublethal concentrations of GNE and/or BMS for 7 days (suppl. Table 2). Cell number was determined by flow-cytometry using lymphocyte gating. Shown are mean ± SD, $n = 4$. **(C)** Colorectal cancer cells were collected, virally transduced to express a Luciferase-P2A-GFP reporter gene and grown to organoids. Following mono- and combination treatment with sublethal concentrations of GNE and BMS for 6 days (concentrations listed in suppl. Table 2), with a repeated treatment after 3 days, viability was assessed using the ONE-GloEX assays. Created in BioRender. Schmitz, L. (2025) https://BioRender.com/uxffdm9. **(D)** Results from the experiments schematically displayed in (C) are shown. Shown are mean ± SD, $n = 2$, two-way ANOVA with Šídák's multiple comparisons test. All values were normalized to their respective DMSO vehicle control.

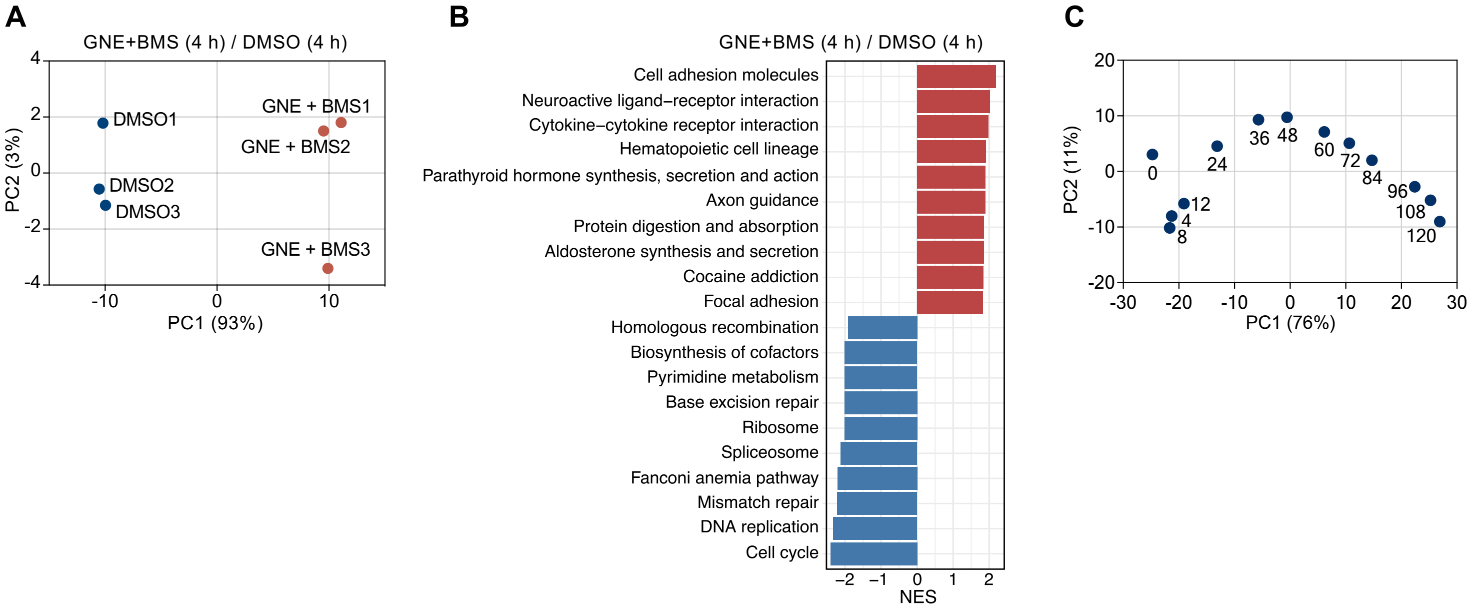

A
GNE+BMS (4 h) / DMSO (4 h)
DMSO1
DMSO2
DMSO3
GNE + BMS1
GNE + BMS2
GNE + BMS3
PC1 (93%)
PC2 (3%)
B
GNE+BMS (4 h) / DMSO (4 h)
Cell adhesion molecules
Neuroactive ligand–receptor interaction
Cytokine–cytokine receptor interaction
Hematopoietic cell lineage
Parathyroid hormone synthesis, secretion and action
Axon guidance
Protein digestion and absorption
Aldosterone synthesis and secretion
Cocaine addiction
Focal adhesion
Homologous recombination
Biosynthesis of cofactors
Pyrimidine metabolism
Base excision repair
Ribosome
Spliceosome
Fanconi anemia pathway
Mismatch repair
DNA replication
Cell cycle
NES
C
PC1 (76%)
PC2 (11%)

D

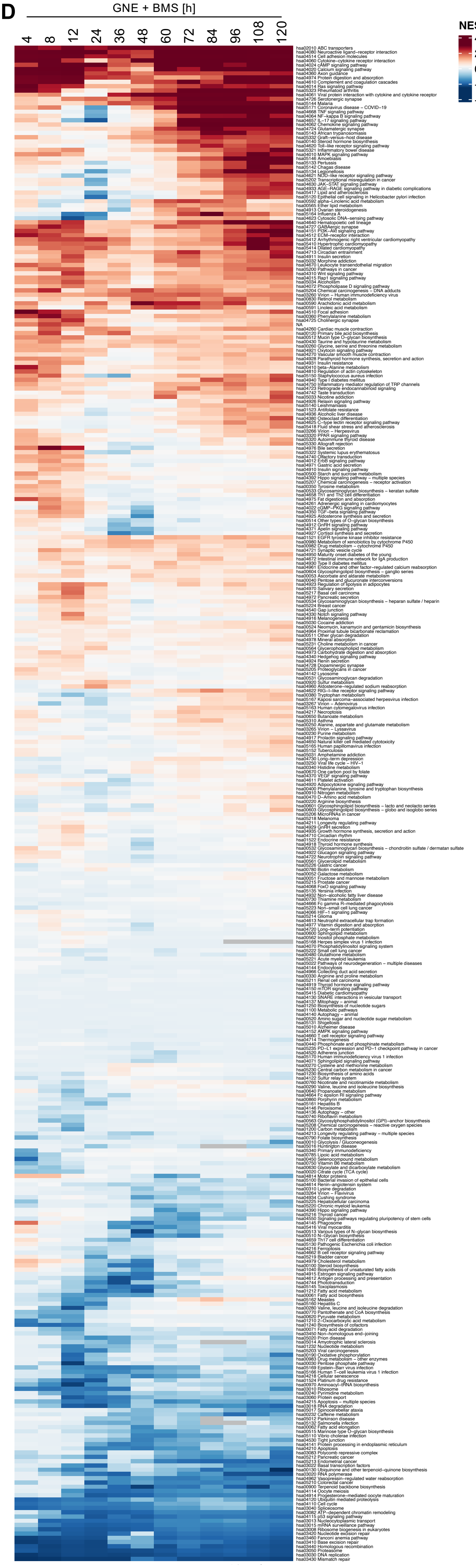
GNE + BMS [h]
4
8
12
24
36
48
60
72
84
96
108
120
NES
4
2
0
−2
−4

E

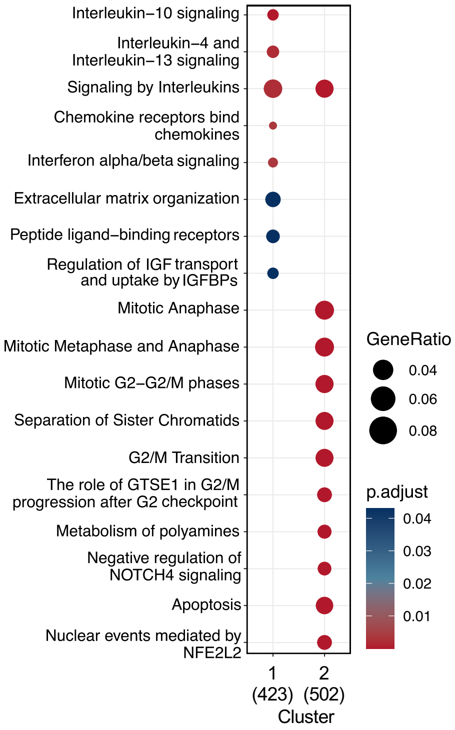
Interleukin−10 signaling
Interleukin−4 and Interleukin−13 signaling
Signaling by Interleukins
Chemokine receptors bind chemokines
Interferon alpha/beta signaling
Extracellular matrix organization
Peptide ligand−binding receptors
Regulation of IGF transport and uptake by IGFBPs
Mitotic Anaphase
Mitotic Metaphase and Anaphase
Mitotic G2−G2/M phases
Separation of Sister Chromatids
G2/M Transition
The role of GTSE1 in G2/M progression after G2 checkpoint
Metabolism of polyamines
Negative regulation of NOTCH4 signaling
Apoptosis
Nuclear events mediated by NFE2L2
1
(423)
2
(502)
Cluster
GeneRatio
0.04
0.06
0.08
p.adjust
0.04
0.03
0.02
0.01

F

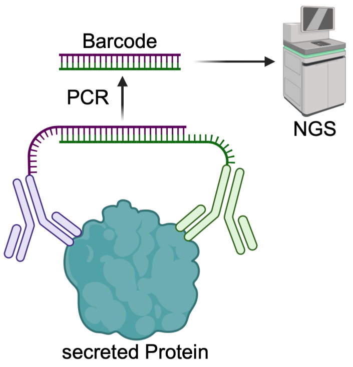
Olink
Barcode
PCR
NGS
secreted Protein

G

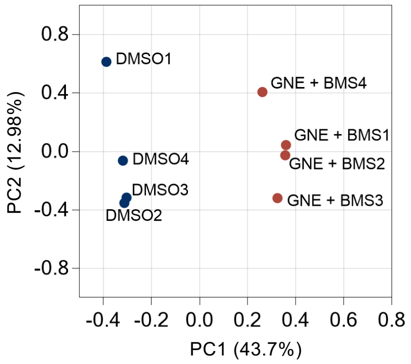
DMSO1
DMSO4
DMSO3
DMSO2
GNE + BMS4
GNE + BMS1
GNE + BMS2
GNE + BMS3
0.8
0.4
0.0
−0.4
−0.8
−0.4
−0.2
0.0
0.2
0.4
0.6
0.8
PC2 (12.98%)
PC1 (43.7%)

Supplementary Data Fig. 3. Characterization of GNE/BMS-mediated effects on RNA expression and protein secretion.

(A) $KRAS^{G12V}$/MYC cells were treated for 4 hours with GNE/BMS or vehicle (DMSO). A principal component (PC) analysis of RNA-seq data shows the first two components, samples are represented by dots, $n = 3$. **(B)** GSEA using the KEGG database of differentially expressed genes (Cut-off: $\log_2 FC \geq 1, \leq -1$; FDR ≤ 0.05). **(C)** PC analysis of the longitudinal RNA-seq experiment over 120 hours post GNE/BMS treatment (Fig. 3B). **(D)** Heatmap displaying all KEGG pathways and their respective normalized enrichment score (NES) values over time. **(E)** GSEA of cluster 1 and 2 (Fig. 3B) using the Reactome database. **(F)** Schematic display of the workflow for the Olink® analysis of secreted proteins. Created in BioRender. Schmitz, L. (2025) https://BioRender.com/vry0ls6. **(G)** $KRAS^{G12V}$/MYC cells were treated for 4 days with GNE/BMS or DMSO. Supernatants were analyzed for secreted proteins using Olink®, a principal component analysis is shown, $n = 4$.

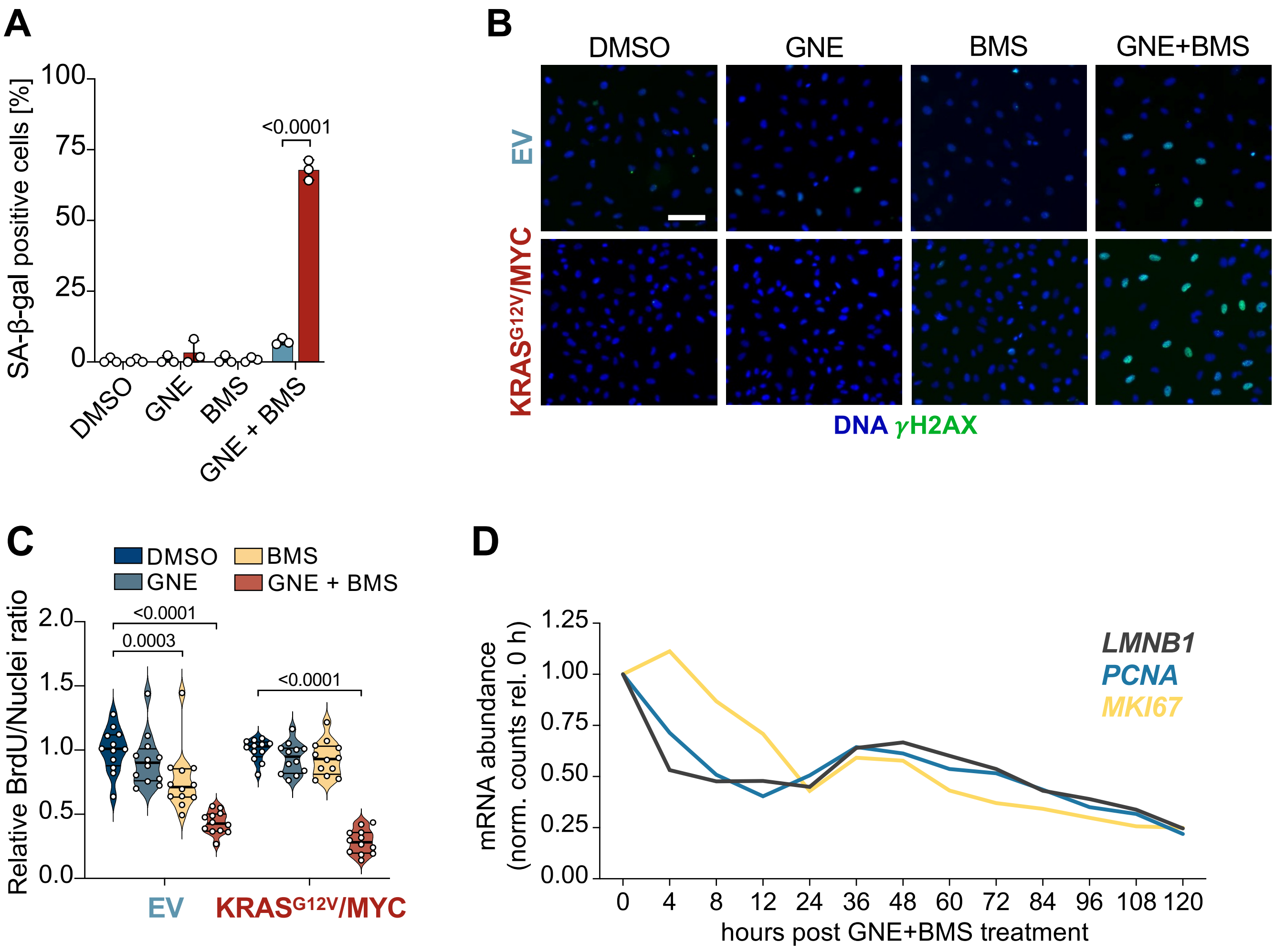

Supplementary Data Fig. 4. The combination of GNE and BMS induces cancer cell-specific senescence.

(A) EV and KRAS^{G12V}/MYC cell lines were analyzed for their SA-ß-gal activity after 3 days of GNE and BMS mono- or combination treatment. SA-ß-gal positive cells were manually counted and normalized to the total cell number per image, n = 3, two-way ANOVA with Šídák's multiple comparisons test. **(B)** Representative γH2AX immunofluorescence images of EV control and KRAS^{G12V}/MYC cancer cell lines after 2 days of GNE and BMS mono- or combination treatment. Scale bar = 100 µm. **(C)** Quantification of BrdU positive cells using immunofluorescence imaging after 2 days of GNE and BMS mono- or combination treatment. Shown are violin plots, n = 4, two-way ANOVA with two-stage linear step-up procedure of Benjamini, Krieger and Yekutieli. **(D)** Expression changes (Deseq2 normalization) of the indicated senescence-associated transcripts *MKI67*, *PCNA* and *LMNB1* (encoding Ki67, PCNA and Lamin B1) were derived from the RNA-seq data and normalized to the expression in untreated cells.

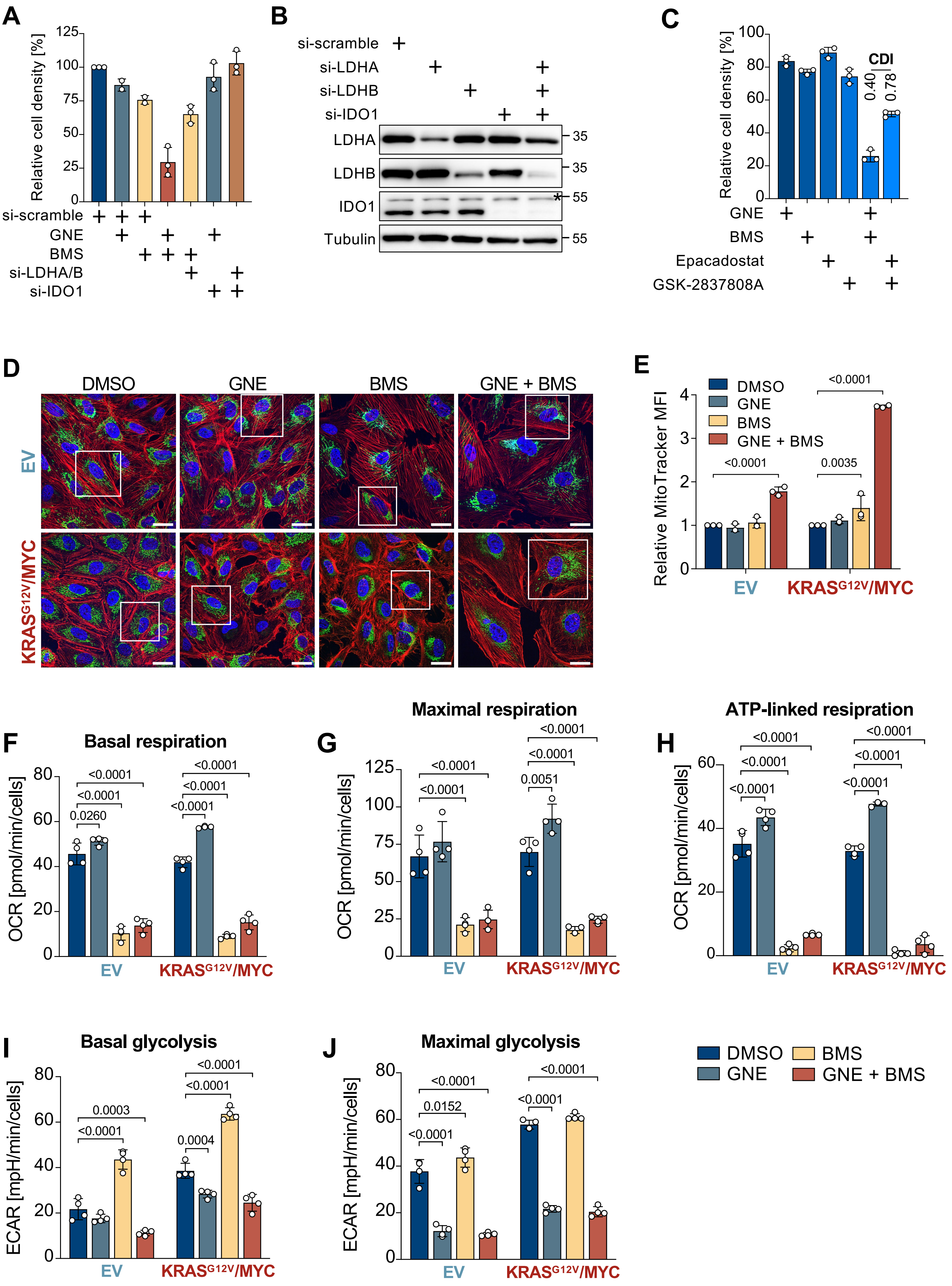
A
Relative cell density [%]
125
100
75
50
25
0
si-scramble
GNE
BMS
si-LDHA/B
si-IDO1
B
si-scramble
si-LDHA
si-LDHB
si-IDO1
LDHA
LDHB
IDO1
Tubulin
35
35
55
55
*
C
Relative cell density [%]
100
80
60
40
20
0
CDI
0.40
0.78
GNE
BMS
Epacadostat
GSK-2837808A
D
DMSO
GNE
BMS
GNE + BMS
EV
KRASG12V/MYC
E
DMSO
GNE
BMS
GNE + BMS
Relative MitoTracker MFI
4
3
2
1
0
<0.0001
<0.0001
0.0035
EV
KRASG12V/MYC
F
Basal respiration
OCR [pmol/min/cells]
80
60
40
20
0
<0.0001
<0.0001
0.0260
<0.0001
<0.0001
<0.0001
EV
KRASG12V/MYC
G
Maximal respiration
OCR [pmol/min/cells]
125
100
75
50
25
0
<0.0001
<0.0001
<0.0001
<0.0001
0.0051
EV
KRASG12V/MYC
H
ATP-linked resipration
OCR [pmol/min/cells]
60
40
20
0
<0.0001
<0.0001
<0.0001
<0.0001
<0.0001
<0.0001
EV
KRASG12V/MYC
I
Basal glycolysis
ECAR [mpH/min/cells]
80
60
40
20
0
0.0003
<0.0001
<0.0001
<0.0001
0.0004
EV
KRASG12V/MYC
J
Maximal glycolysis
ECAR [mpH/min/cells]
80
60
40
20
0
<0.0001
0.0152
<0.0001
<0.0001
<0.0001
EV
KRASG12V/MYC
DMSO
BMS
GNE
GNE + BMS

Supplementary Data Fig. 5. Mitochondrial effects of BMS.

KRAS^{G12V}/MYC cells were transfected with siRNAs targeting either LDHA/B or IDO1 alone or combined with GNE and/or BMS treatment as shown. Transfection was repeated after 2 days. **(A)** After 4 days one half of the cells was analyzed for effects on cell density, values were normalized to si-scramble, shown are mean ± SD, *n* = 3. **(B)** The other half of the siRNA-treated cells was analyzed by Western blotting for knock-down, the asterisk indicates a non-specific band, the positions of molecular weight markers are indicated. **(C)** Cells were treated for 3 days with Epacadostat (IDO1 inhibitor, 40 μM), GSK-2837808 (LDHA/B inhibitor, 35 μM) alone or in combination with GNE or BMS, as shown. Cell viability was determined, shown are mean ± SD, *n* = 3. The Coefficient of Drug Interaction (CDI) for GNE and BMS as well as Epacadostat and GSK-2837808 was calculated and indicated above respective bar plots. **(D)** COX8-GFP EV and KRAS^{G12V}/MYC cells were treated for 2 days with the indicated conditions and stained with Hoechst33342 (nuclear DNA) and Phalloidin (Actin-filaments) following immunofluorescence analysis. White rectangular selections are highlighting the cells used for mitochondrial high-resolution 3D-rendering (Fig. 5A). **(E)** Cells treated with GNE and/or BMS for 2 days, loaded with MitoTracker Deep Red FM (200 nM for 15 minutes) and subsequently analyzed using flow-cytometry. Shown are median fluorescence intensities (MFI), *n* = 3, two-way ANOVA with Dunnet's multiple comparisons test. The next subfigures show seahorse metabolic flux analyses of empty vector and KRAS^{G12V}/MYC cell lines using the MitoStress Test assay after 4 hours of GNE and BMS mono- and combination treatment using Oligomycin (2 μM), FCCP (2 μM) and Rotenone/Antimycin A (0.5 μM). **(F-H)** Analysis of basal, maximal and ATP-linked respiration. **(I, J)** Quantification of basal and maximal glycolysis. Shown are mean ± SD, *n* = 4, two-way ANOVA with Dunnet's multiple comparisons test.
