# Supplementary material for: Synergistic targeting of cancer cells through simultaneous inhibition of key metabolic enzymes
Source: Cell Death Differ. 2025 Jun 23;32(12):2239–56. doi: 10.1038/s41418-025-01532-5 (PMC12669732; doi:10.1038/s41418-025-01532-5)
Supplement: Supplementary file 2 — Supplementary Table 1 [file 41418_2025_1532_MOESM2_ESM.pdf]

Supplementary Table 1. Inhibitors used in synthetic lethal screen

Name	Target	Sublethal concentration	Source	Cat #
(±)-3-Methyl-2-oxovaleric acid	KGDHC	1 mM	Sigma	K7125
(R)-GNE-140	LDHA/B	7.5 μM	MedChemExpr	HY-100742A
AZD-3965	MCT1	5 μM	SellekChem	S7339
BCH	LAT1	10 μM	Tocris	5027
BMS-986205	IDO1	6 μM	MedChemExpr	HY-101560
CB-839	GLS	10 μM	SellekChem	S7655
Cytochalasin B	GLUT	0.2 μM	Santa Cruz	sc-3519
Dichloracetate	PDK	1 mM	Sigma	347795
Eflornithin	ODC	60 μM	Tocris	2761
Etomoxir	CPT1	50 μM	Sigma	S8244
H3B-120	CPS1	20 μM	Sigma	SML3007
Indoximod	IDO/TDO	2 μM	SelleckChem	S7756
Metformin	Complex I	10 mM	Invivogen	NC2257632
PFK15	PFKFB3	0.75 μM	Sigma	S7289
TVB-2640	FASN	0.3 μM	Sigma	S9714
V-9302	ASCT2	3 μM	MedChemExpr	HY-112683
α-cyano-4-hydroxycinnamic acid	MCT1	1 mM	Sigma	C2020
