# Supplementary material for: Synergistic targeting of cancer cells through simultaneous inhibition of key metabolic enzymes
Source: Cell Death Differ. 2025 Jun 23;32(12):2239–56. doi: 10.1038/s41418-025-01532-5 (PMC12669732; doi:10.1038/s41418-025-01532-5)
Supplement: Supplementary file 3 — Supplementary Table 2 [file 41418_2025_1532_MOESM3_ESM.pdf]

Supplementary Table 2. Sublethal concentrations of GNE and BMS for different cell lines

Related Manuscript Figure	Cell line	RRID	Species	(R)-GNE-140 [μM]	BMS-986205 [μM]
Figure 2A	FS4-LTM (from FS4)	CVCL_0266	Human	14	4
	HEK293T	CVCL_0063	Human	30	2.5
	HCT-116	CVCL_0291	Human	20	8
	HeLa	CVCL_0030	Human	15	10
	HT-29	CVCL_0320	Human	20	6
	iFTSEC (hTERT FT240)	CVCL_UH60	Human	7.5	6
	iFTSEC_EV	This study	Human	7.5	6
	iFTSEC_KRASG12V/MYC	This study	Human	7.5	6
	KPC	CVCL_A9ZK	Mouse	20	9
	LN229	CVCL_0393	Human	20	20
	MCF-7	CVCL_0031	Human	15	6
	mPSC4	N/A	Mouse	10	1
	OVCAR-4	CVCL_1627	Human	7.5	6
	PancOVA (from Panc02)	CVCL_D627	Mouse	24	9
	S2-007	CVCL_B279	Human	26	6
	PC9	CVCL_B260	Human	15	8
	RPE-1	CVCL_4388	Human	8	15
	SW620	CVCL_0547	Human	10	6
	T110299	N/A	Mouse	26	10
	U2OS	CVCL_0042	Human	30	10
Figure 2B	MYC/BCL2 H5071	N/A	Human	30	6
	MYC/BCL2 H3388	N/A	Human	20	3
	MYC/BCL2 H3907	N/A	Human	20	4
	BCL6/BCL2 H5071	N/A	Human	20	4.5
	BCL6/BCL2 H3388	N/A	Human	15	3
	BCL6/BCL2 H3907	N/A	Human	20	4.5
Figure 2D	O06	N/A	Human	50	10.8
	O20	N/A	Human	23.2	10.8
	O15	N/A	Human	23.2	10.8
	O05	N/A	Human	23.2	5
	O04	N/A	Human	50	10.8
	O16	N/A	Human	23.2	2.3
	O09	N/A	Human	23.2	10.8
