# Supplementary material for: Synergistic targeting of cancer cells through simultaneous inhibition of key metabolic enzymes
Source: Cell Death Differ. 2025 Jun 23;32(12):2239–56. doi: 10.1038/s41418-025-01532-5 (PMC12669732; doi:10.1038/s41418-025-01532-5)
Supplement: Supplementary file 5 — Supplementary Table 4 [file 41418_2025_1532_MOESM5_ESM.pdf]

Supplementary Table 4. Mutations and copy number variations for the cancer genes showing significant enrichment.

Gene	Cell line	Protein change	Mutation type	Mutation zygosity
EP300	BxPC-3	p.R397*	Substitution - Nonsense	het
EP300	DLD-1	p.R838C	Substitution - Missense	het
EP300	DLD-1	p.E1014*	Substitution - Nonsense	het
EP300	HCT 116	p.M1470fs*26	Deletion - Frameshift	het
EP300	HCT 116	p.N1700fs*9	Deletion - Frameshift	het
EP300	HCT 15	p.E1014*	Substitution - Nonsense	hom
EP300	HT	p.N1547fs*17	Deletion - Frameshift	het
EP300	LS411N	p.H2324fs*55	Insertion - Frameshift	het
EP300	MCF-7	p.R1356*	Substitution - Nonsense	het
EP300	MOLT-4	p.M1470fs*26	Deletion - Frameshift	het
EP300	RKO	p.K292fs*25	Deletion - Frameshift	het
EP300	RKO	p.M1470fs*26	Deletion - Frameshift	het
EP300	RL	p.Y1414C	Substitution - Missense	het
EP300	RL	p.E1011*	Substitution - Nonsense	het
EP300	RL95-2	p.H2324fs*55	Insertion - Frameshift	het
EP300	SU-DHL-6	p.R1627W	Substitution - Missense	het
EP300	SW48	p.M1470fs*26	Deletion - Frameshift	het
EP300	SW620	p.P1440L	Substitution - Missense	het
EP300	T24	p.C1201Y	Substitution - Missense	hom
LRP1B	Hs 766T		Gene deletion	
LRP1B	NCI-H460		Gene deletion	
LRP1B	OVCAR-3		Gene deletion	
LRP1B	U2OS		Gene deletion	
RB1	5637	p.Y325*	Substitution - Nonsense	hom
RB1	BT-20	p.P515L	Substitution - Missense	het
RB1	BT-549		Gene deletion	
RB1	DU145	p.K715*	Substitution - Nonsense	hom
RB1	DU4475		Gene deletion	
RB1	TCCSUP		Gene deletion	
