# Supplementary material for: Synergistic targeting of cancer cells through simultaneous inhibition of key metabolic enzymes
Source: Cell Death Differ. 2025 Jun 23;32(12):2239–56. doi: 10.1038/s41418-025-01532-5 (PMC12669732; doi:10.1038/s41418-025-01532-5)
Supplement: Supplementary file 6 — Original Western blots [file 41418_2025_1532_MOESM6_ESM.pdf]

Figure 4 M

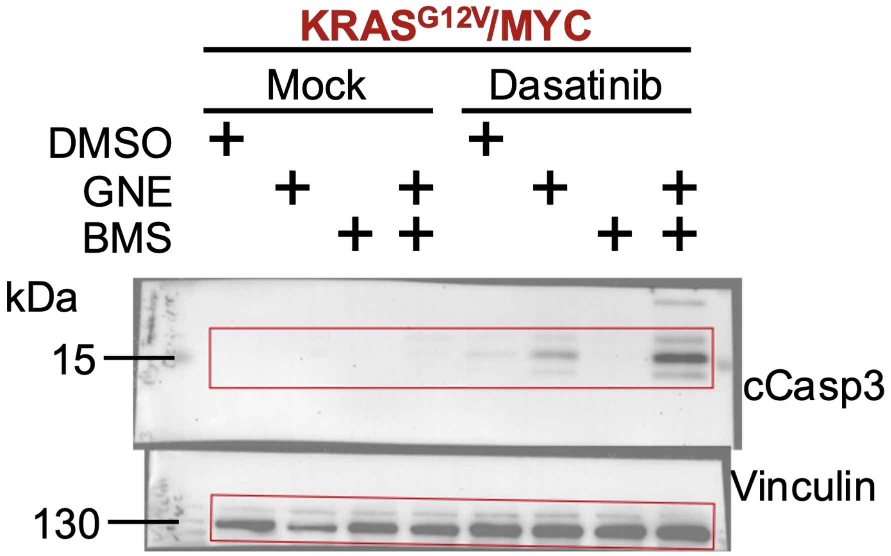

Rectangular selection marks regions included in the figure.

Figure 7 D

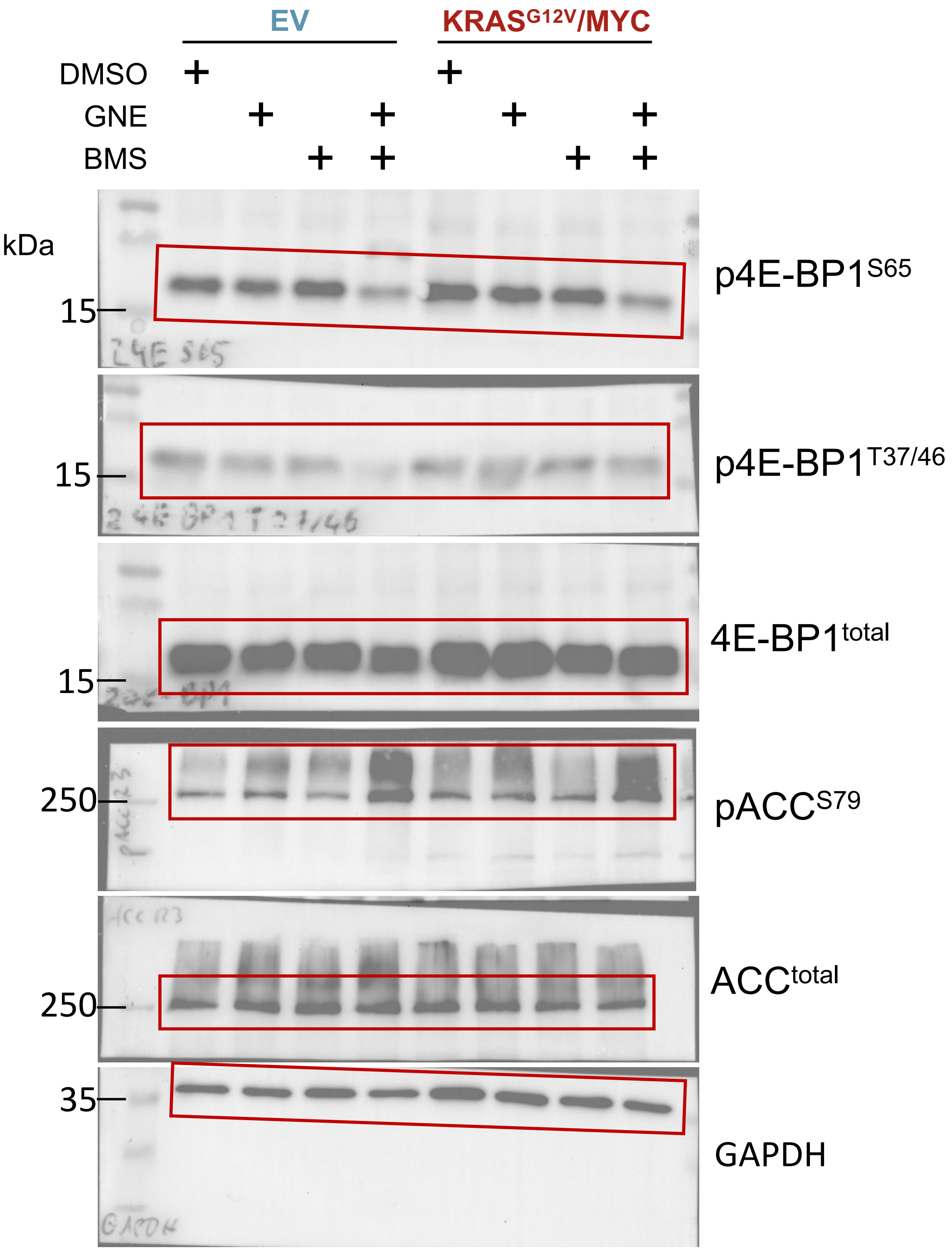

Rectangular selection marks regions included in the figure.

Supplementary Figure 5 B

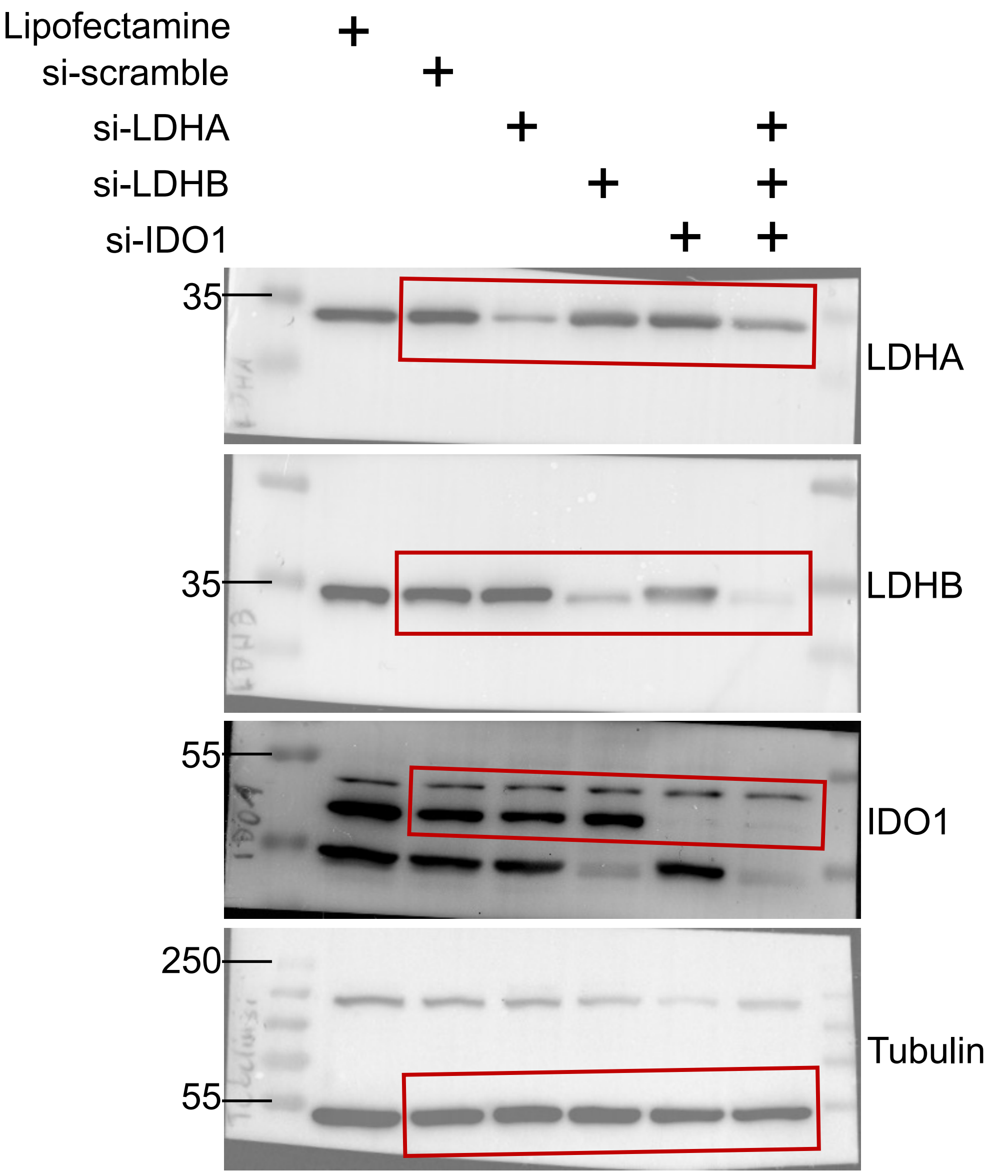

Rectangular selection marks regions included in the figure.
